# Application of Machine-Learning Models to Predict Tacrolimus Stable Dose in Renal Transplant Recipients

**DOI:** 10.1038/srep42192

**Published:** 2017-02-08

**Authors:** Jie Tang, Rong Liu, Yue-Li Zhang, Mou-Ze Liu, Yong-Fang Hu, Ming-Jie Shao, Li-Jun Zhu, Hua-Wen Xin, Gui-Wen Feng, Wen-Jun Shang, Xiang-Guang Meng, Li-Rong Zhang, Ying-Zi Ming, Wei Zhang

**Affiliations:** 1 Department of Clinical Pharmacology, Xiangya Hospital, Central South University, Changsha, 410008, Hunan, P. R. China; 2 Institute of Clinical Pharmacology, Central South University; Hunan Key Laboratory of Pharmacogenetics, Changsha, 410078, Hunan, P. R. China; 3 Peking University Third Hospital, Beijing, 100191, P. R. China; 4 Research Center of Chinese Health Ministry of Transplantation Medicine Engineering and Technology, Third Affiliated Hospital, Central South University, Changsha, 410013, Hunan, P. R. China; 5 Department of Clinical Pharmacology, Wuhan General Hospital of Guangzhou Command, Wuhan, 430070, Hubei, P. R. China; 6 Department of Renal Transplantation, The First Affiliated Hospital of Zhengzhou University, Zhengzhou, 450052, Henan, P. R. China; 7 School of Basic Medical Sciences, Zhengzhou University, Zhengzhou, 450001, Henan, P. R. China

## Abstract

Tacrolimus has a narrow therapeutic window and considerable variability in clinical use. Our goal was to compare the performance of multiple linear regression (MLR) and eight machine learning techniques in pharmacogenetic algorithm-based prediction of tacrolimus stable dose (TSD) in a large Chinese cohort. A total of 1,045 renal transplant patients were recruited, 80% of which were randomly selected as the “derivation cohort” to develop dose-prediction algorithm, while the remaining 20% constituted the “validation cohort” to test the final selected algorithm. MLR, artificial neural network (ANN), regression tree (RT), multivariate adaptive regression splines (MARS), boosted regression tree (BRT), support vector regression (SVR), random forest regression (RFR), lasso regression (LAR) and Bayesian additive regression trees (BART) were applied and their performances were compared in this work. Among all the machine learning models, RT performed best in both derivation [0.71 (0.67–0.76)] and validation cohorts [0.73 (0.63–0.82)]. In addition, the ideal rate of RT was 4% higher than that of MLR. To our knowledge, this is the first study to use machine learning models to predict TSD, which will further facilitate personalized medicine in tacrolimus administration in the future.

Tacrolimus is one of the most widely used immunosuppressive agents to prevent acute rejection following solid organ transplantation. More than 70% of renal transplant patients received this effective agent in 2004^1^. However, the use of tacrolimus has to be cautious due to its narrow therapeutic index and remarkable variability of inter- and intra-individual bioavailabilities^2^. Insufficient dosing of tacrolimus is associated with an increased risk for acute rejection^3^, while overexposure with higher rate of drug-related toxicities, such as nephrotoxicity, neurotoxicity, and new-onset diabetes^4^,^5^. Daily monitoring and maintaining target concentration is important to decrease allograft rejection and toxicity^6^. Therefore, there is an increasing need to develop improved strategies for determining the appropriate dose in clinic.

Many factors affecting the pharmacokinetics of tacrolimus have been identified, including clinical factors such as ethnicity, age, gender, concomitant medication, hepatic and renal dysfunction, and genetic factors such as *CYP3A5, CYP3A4* and *ABCB1* single nucleotide polymorphisms (SNPs)^7^,^8^. Among these factors, *CYP3A5* genotype is associated with a remarkable impact on the tacrolimus pharmacokinetics, while the effects of other genetic polymorphisms are rather limited or conflicting^9^,^10^,^11^. A number of algorithms containing clinical and/or pharmacogenomic factors have been constructed to predict tacrolimus dose; meanwhile, retrospective and prospective trials have been conducted to verify these algorithms^9^,^12^,^13^,^14^,^15^,^16^,^17^,^18^,^19^. Thervet *et al*. demonstrated that *CYP3A5* genotype guided tacrolimus dosing enabled more renal recipients to achieve target tacrolimus trough (C0) levels after three days of tacrolimus treatment. In addition, these patients took less time to reach their target concentration with fewer dose modifications^12^. A more recent trial indicated that CYP3A4 activity and *CYP3A5* genotype can explain 56–59% variability in tacrolimus dose and clearance^9^. These successes in clinic revealed the possibility to improve clinical outcomes of tacrolimus therapy by taking pharmacogenomic factors into account.

However, most of the algorithms for predicting tacrolimus dose were based on relatively small clinical population, and the predictive accuracy was usually uncertain. Moreover, proposed algorithms are mostly based on multiple linear regression (MLR) methods, which have some well-known limitations that may impair prediction accuracy. For example, MLR model assumes independence between variants, and the relationship between the dependent and independent variables is always complex and non-linear^20^. Therefore, MLR may not be the most applicable model for accurate prediction of drug outcomes.

Machine learning techniques, compared with traditional statistical models, have many advantages including high power and accuracy, ability to model non-linear effects, interpretation of large genomic data sets, robustness to parameter assumptions and dispense with normal distribution test^21^. Recently it has been widely used in predicting warfarin dose^22^. For example, Random Forest Regression (RFR), Boosted Regression Tree (BRT) and Support Vector Regression (SVR) models were utilized to predict warfarin maintenance dose in African Americans, with much higher accuracies than previous models^21^. Artificial neural networks (ANNs) algorithm reached high accuracy in predicting warfarin maintenance dose, more than 70% of patients in the low (≤21 mg/) and median dose (21–49 mg) subgroups have been correctly identified^23^. In our previous work, eight machine learning algorithms were compared with MLR in predicting warfarin dosing, results showing Bayesian additive regression trees (BART), multivariate adaptive regression splines (MARS) and SVR significantly outperformed other models; machine learning methods also performed better than MLR in the low- and high- dose ranges^20^.

To our knowledge, the development and application of machine learning algorithms to predict tacrolimus dose has not been reported. We therefore conducted this research to investigate the clinical and genetic factors significantly associated with tacrolimus stable dose as well as to identify the most feasible algorithm for prediction of the dose requirement of Chinese renal transplantation recipients.

## Results

### Basic Characteristics of the Study Cohorts

In total, 1,045 renal transplant recipients were enrolled in the trial, whose basic characteristics are shown in Table 1. Continuous variables are shown as mean ± standard deviation, and categorical variables are shown as number (%). No significant difference was found in the demographic, clinical and genetic data between the derivation cohort (n = 838) and the validation cohort (n = 207). All the tested SNPs were in Hardy–Weinberg equilibrium except *ABCB1*129TC and *ABCB1*2677GTA, and the genotype frequencies were in accordance with previously reported data of Chinese population (Supplementary Table S1).

In the derivation cohort, the mean TSD among these patients was 3.48 ± 1.28 mg/day. Patients had an average age of 36.19 ± 10.62 years old, and 71.2% were males. The most common complication was hypertension with 561 patients (66.9%), while 19 patients were diagnosed diabetes (2.3%). The most common combined medication was calcium channel blocker (544 patients, 64.9%), in addition, 377 patients were given metoprolol (45.0%), and 246 were given omeprazole (29.4%). Among all these patients, 50.6% were carriers of CYP3A5*3 GG genotype, 9.2% were carriers of AA genotype, and 16 unknown (1.9%). The detected results of other genotypes are illustrated in Supplementary Table S1.

Patients in the validating cohort exhibited similar results in the TSD (3.50 ± 1.32 mg/day), age (35.82 ± 10.34) and sex (71.5% were males). Comparably, 134 (64.7%) patients had hypertension, and 127 (61.4%) patients were given calcium channel blocker. Genotyping data showed that 46.9% of patients were carriers of CYP3A5*3 GG genotype, 7.2% were AA genotype, with 8 (3.8%) unknown genotype (Table 1).

### Identification of Clinical and Genetic Factors Significantly Associated with TSD

In order to construct a predictive model that only contains important factors, we have first investigated the relationship between each factor and the STD of patients. Univariate analysis was used to test all the clinical and genetic factors, resulted in four factors that were significantly associated with TSD: whether has hypertension, whether has diabetes, whether taking omeprazole and *CYP3A5* genotype. Among them, *CYP3A5*6986AG is the most significant influencing factor, with a *P* value of 2.2*10^−16^ in the F-test (Supplementary Table S2).

Tacrolimus stable dose was also compared among patients with different *ABCB1* genotypes. The comparison was carried out for the three polymorphisms (1236 C/T, 2677 G/A/T and 3435 C/T) most studied in the literature. No statistically significant difference was found (data not shown).

Next, multivariable regression model was used to test the above four variates. It was shown that diabetes was not a significant factor in the model and thereafter excluded. The remaining three factors (hypertension, use of omeprazole and *CYP3A5* genotype) were used to construct the MLR and 8 machine learning models. (Supplementary Table S3).

### Overall Comparison of Predictive Algorithms

**Figure 1:**
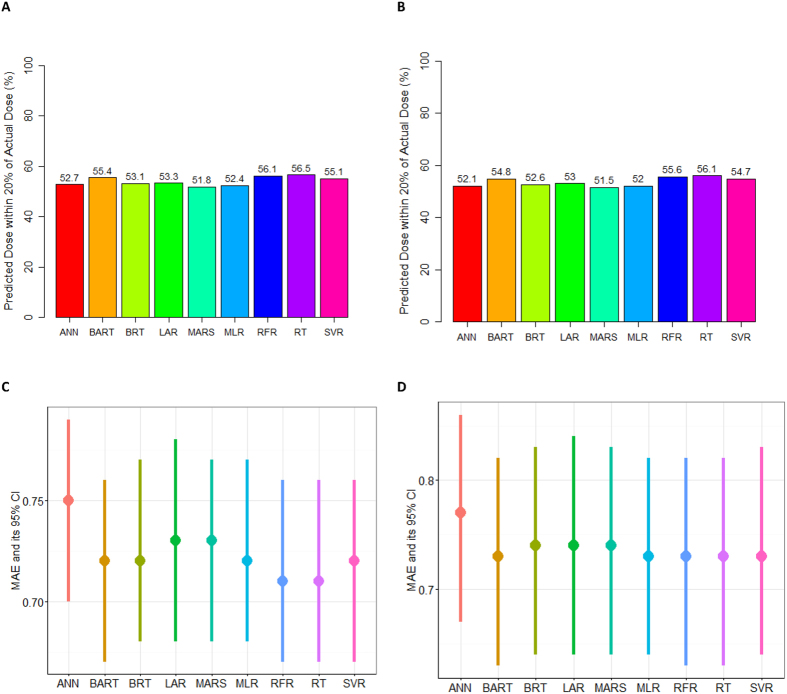
Ideal rate and mean absolute error in train and test partitions for nine techniques averaged over 100 round of resampling process results for different models fitted. Predicted dose within 20% of the actual dose in the train (**A**) and test (**B**) set of the derivation cohort. Mean absolute error between the predicted and actual dose in the train (**C**) and test (**D**) set. The vertical bars represent the 95% CIs of MAE. MLR: multiple linear regression; SVR: support vector regression; ANN: artificial neural network; RT: regression tree; RFR: random forest regression; BRT: boosted regression tree; MARS: multivariate adaptive regression splines; LAR: lasoo regression; BART: Bayesian additive regression trees.

**Figure 2:**
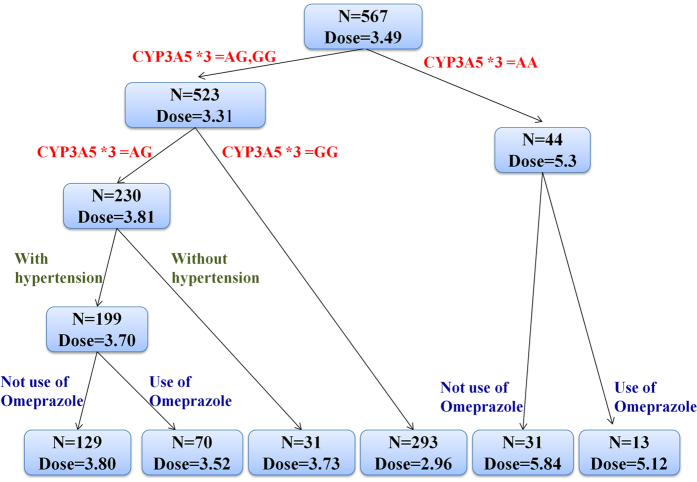
Predicted tacrolimus doses according to the regression tree algorithm. N and dose represent the sample size and predicted tacrolimus dose, respectively.

In order to determine the overall predictive accuracy, approaches including MAE and ideal rate (predictive dose fell within 20% of the actual dose) were applied. Among all the machine learning models, RT performed best in both derivation [0.71 (0.67–0.76)] and validation cohorts [0.73 (0.63–0.82)] (not statistically significant) (Fig. 1). Although MLR had similar MAE with RT, the ideal rate of RT was 4% higher than that of MLR. On the other hand, the lowest MAE was also seen using RFR model. However, as RT is a simpler and more easily understood model that fits clinical use, it was chosen for this study. The developed RT model is shown as Fig. 2.

### Clinical Relevance

In general, RT algorithm provided more accurate prediction of TSD than other 8 algorithms. The performance of the three dose ranges, low dose (≤2.5 mg/day), intermediate dose (2.5–4 mg/day), and high dose (>4 mg/day), were compared as shown in Tables 2 and 3. Patient doses in the intermediate range were best predicted compared with the actual stable dose in both derivation and validation cohorts (MAE = 0.50 and 0.48 mg/day, respectively) (Table 2). For patients who required 2.5 mg/day or less (24.4% of the total 693 patients), 38.5% of the predicted dosage fell into ideal dose range (20% of the actual dose). While for the patients who required 4 mg/day or more (20.8% of the total patients), 44.4% of the prediction dropped into ideal dose.

## Discussion

Compared with traditional dosing strategies in clinic, the current study was successful in providing a novel approach that can predict TSD more accurately and conveniently. In general, the performances of the 9 algorithms were similar in predicting TSD. While the best performance was observed in RT model in this study, comprehensive evaluation of these algorithms in various studies is needed to come to a final conclusion. It should also be noted that the current study was performed in Chinese, studies in other ethnic groups may come to different results.

The most influential factor in this study was *CYP3A5* genotype. The SNP 6986 A > G on the *CYP3A5* gene results in absence of function protein. Carriers of homozygous 6986 G allele (designated as CYP3A5*3) have no CYP3A5 activity, which impair the whole-blood concentration of tacrolimus^24^ and subsequently the time required to reach target concentration^3^. None of the included *ABCB1* SNPs were found any significant impact on the algorithm. In fact, previous researches checking the association between *ABCB1* genotypes and tacrolimus dosage have come to conflicting results^24^,^25^,^26^.

Our results indicated that the intermediate dose range exhibited better accuracy (lower MAE and higher ideal rate) than that in the high- and low- dose ranges. Nevertheless, patients in this dose range are less likely to benefit from statistical models based on pharmacogenomics. In practice, patients who require extreme dose administrations (or whom grouped in the high- and low- dose ranges) are more likely to face overdose or underdose and hence suffer from adverse clinical consequence^22^. Therefore, better prediction of extreme dose ranges are needed to present real help to those patients.

Whilst machine learning techniques demonstrated their capability in solving inferential problems by self-adjust their structure when encounter errors, as well as dealing with numerous variables simultaneously^20^, we should be noted that they are still far from omnipotent in clinical use. The relationship between dependent variables and independent variables are very complicated in all these statistical algorithms, and the existence of gene-gene and gene-environment interactions bring more challenge to the researchers^27^,^28^,^29^. Inclusion of larger number of genotypic variables in a predictive model may be helpful to obtain a better performance, but this may lead to addition of redundant data and may hinder its application in clinical practice^21^. The complicated situation of real patients should be well considered, as additional comorbidity and interacting drugs are always the case, which may not be completely included in the models^30^. Therefore, even the statistical models are utilized to increase the predictive accuracy of TSD, continuous monitoring of drug concentration is still needed at the moment.

There are some limitations in this study, no other potentially important factors were included, such as smoking, alcohol consumption and other genetic factors; secondly, data regarding tacrolimus initial doses or adverse reactions were not gathered in the study, only data about stable therapeutic doses were considered; in addition, using of *p*-value threshold to select significant SNPs may be not enough to generate most complementary SNP set^21^.

## Methods

### Patients

Stable tacrolimus-treated renal recipients at The Third Xiangya Hospital of Central South University and Peking University Health Science Center between Oct 2012 and Sep 2014 were considered for enrollment. All patients were Chinese with a minimum age of 18 years old. The clinical research admission was approved by Chinese Clinical Trial Registry (registration number: ChiCTR-RNC-12002894). The study protocol was approved by the Ethics Committee of Institute of Clinical Pharmacology, Central South University (CTXY-120030-2), all methods were performed in accordance with the relevant guidelines and regulations, and written informed consent was obtained from all patients.

The demographic and clinical information of the subjects were obtained from their clinical records as well as clinical and telephone follow-ups. Information of combined diseases such as hypertension and diabetes and concomitant medications such as omeprazole, metoprolol and calcium channel blockers were collected. All the patients received tacrolimus, together with mycophenolate and glucocorticoid after transplantation. Tacrolimus was initiated at 0.05 mg/Kg every 12 h then dose adjusted to target trough concentration of 6–10 ng/ml for the first month, and then 6–8 ng/ml afterwards. Tacrolimus concentration was monitored daily during the hospital stay and in the follow-up visits. Doses were adjusted by 25% each time when it was out of the above target range. Tacrolimus stable dose (TSD) was defined as the total daily dose after 3 months of transplantation, and at least three consecutive blood concentrations were within target range and within 20% of each other^19^.

### Genotyping

MassARRAY (Sequenom Inc., CA, USA) was used for genotyping. Detected genes included *CYP3A5* (coding for tacrolimus metabolizing enzymes) and *ABCB1* (coding for the drug transporter p-glycoprotein). Polymorphisms in the *CYP3A5* (6986 A/G) and *ABCB1* (3435 C/T, 129 T/C, 1236 C/T and 2677 G/T/A) were genotyped. The genotyping was verified by repeating 20 random samples by MassARRAY and direct sequencing of 10 random samples with Beckman Coulter CEQ800.

### Model Building and Statistical Analyses

**Figure 3:**
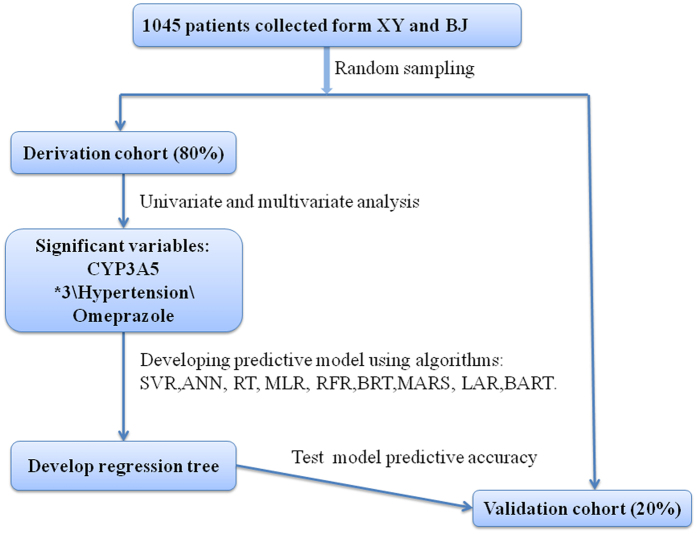
Study flow chart. MLR: multiple linear regression; SVR: support vector regression; ANN: artificial neural network; RT: regression tree; RFR: random forest regression; BRT: boosted regression tree; MARS: multivariate adaptive regression splines; LAR: lasoo regression; BART: Bayesian additive regression trees.

The overall modeling process is illustrated as Fig. 3. Generally, 80% (838 patients) of the eligible patients were randomly selected as the “derivation cohort” to develop dose-prediction algorithm. The remaining 20% of the patients (207 patients) constituted the “validation cohort”, which was used to test the final selected algorithm. Meanwhile, to obtain robust results, 100 times of resampling were run to minimize the overfitting problem. Next, univariate and stepwise multivariate linear regression (MLR) was used to select covariates related to tacrolimus stable dose. Covariates with statistical significance (*CYP3A5* genotype, hypertension and use of omeprazole) were used to develop algorithms within derivation cohort (train set). The performances of the algorithms were evaluated and compared using the mean absolute error (MAE) and the mean percentage of patients whose predicted dose fell within 20% of the actual dose (ideal rate) in the remaining 20% of patients (test set). The MAE is defined as the average of the absolute value of actual dose minus predicted dose, while the percentage of patients within 20% of the actual dose was selected by us since this definition has been widely applied^22^. Descriptive statistics was utilized to determine means and standard deviations, frequency and percentage distributions. Chi-square test was used to assess deviations of allele frequencies from Hardy-Weinberg equilibrium.

MLR and eight machine learning techniques, namely, support vector regression (SVR), artificial neural network (ANN), regression tree (RT), random forest regression (RFR), boosted regression tree (BRT), multivariate adaptive regression splines (MARS), lasoo regression (LAR), Bayesian additive regression trees (BART) were applied in tacrolimus dose prediction. All analyses in this study were implemented using R (Version 3.2.2)^31^ with related packages or our custom written functions. We used the RSNNS package for ANN^32^, rpart package for RT^33^, gbm package for BRT^34^, e1071 package for SVR^35^, randomForest package for RFR^36^, earth package for MARS^37^, glmnet package for LAR^38^ and bartMachine package for BART^39^. Default parameters were used (Supplementary Table S4).

In the validation cohort, the MAE and ideal rate of the pharmacogenomic algorithm were calculated overall, also in terms of tacrolimus dose range, which was divided into three categories based on the 25% and 75% quartiles of TSD: low dose (≤2.5 mg/day), intermediate dose (2.5–4 mg/day), and high dose (>4 mg/day).

## Additional Information

**How to cite this article**: Tang, J. *et al*. Application of Machine-Learning Models to Predict Tacrolimus Stable Dose in Renal Transplant Recipients. *Sci. Rep.* **7**, 42192; doi: 10.1038/srep42192 (2017).

**Publisher's note:** Springer Nature remains neutral with regard to jurisdictional claims in published maps and institutional affiliations.

## Supplementary Material

Supplementary Table S1–S4

Supplementary Table S5

## Figures and Tables

**Table 1:** Basic characteristic of the patients.

Variable	The Derivation Cohort (N = 838)	The Validating Cohort (N = 207)
Continuous variable mean (sd)		
Tacrolimus stable dose − mg/day	3.48 (1.28)	3.50 (1.32)
Age (year)	36.19 (10.62)	35.82 (10.34)
Height− cm	165.36 (7.84)	166.14 (7.53)
Weight − kg	58.43 (11.01)	58.39 (9.81)
Hemoglobin − g/dl	118.29 (26.63)	121.69 (26.06)
Leukocyte	7.63 (2.66)	7.64 (2.87)
Serum creatinine	422.07 (463.78)	429.15 (524.21)
Total bilirubin	11.42 (6.68)	11.28 (4.41)
Albumin − g/l	43.35 (5.47)	44.76 (4.68)
Categorical variable n. (%)		
Male	597 (71.2)	148 (71.5)
Diabetes	19 (2.3)	3 (1.4)
Hypertension	561 (66.9)	134 (64.7)
Living donor	420 (50.1)	107 (51.7)
Anemia	409 (48.8)	94 (45.4)
Cardiac insufficiency	33 (3.9)	11 (5.3)
Use of Calcium channel blocker	544 (64.9)	127 (61.4)
Use of Metoprolol	377 (45.0)	84 (40.6)
Use of Omeprazole	246 (29.4)	59 (28.5)
Use of Furosemide	420 (50.1)	107 (51.7)
ACEI/ARA*	185 (22.1)	43 (20.8)
Cephalosporin	513 (61.2)	126 (60.9)
Infected	190 (22.7)	44 (21.2)
CYP3A5 *3		
A/A	77 (9.2)	15 (7.2)
A/G	321 (38.3)	87 (42.0)
G/G	424 (50.6)	97 (46.9)
Unknown	16 (1.9)	8 (3.8)

^*^ACEI/ARA: Angiotensin converting enzyme inhibition, Angiotensin II receptor antagonist.

**Table 2:** Predicted tacrolimus stable doses with the regression tree algorithm compared with the actual stable dose in the derivation and validation cohorts.

Cohort	Mean Absolute Error (95% CI) mg/day	
	Derivation cohort	Validation cohort
Overall	0.71 (0.66–0.75)	0.73 (0.63–0.84)
≤2.5 mg/day	1.31 (1.20–1.42)	1.33 (1.13–1.53)
>2.5 mg/day to <4 mg/day	0.50 (0.46–0.54)	0.48 (0.39–0.58)
≥4 mg/day	1.07 (0.96–1.18)	1.14 (0.92–1.37)

**Table 3:** Percentage of patients in the validation cohort and in the derivation-plus-validation cohort with an ideal, underestimated, or overestimated dose of tacrolimus in renal transplant patients requiring low, intermediate, or high actual doses of tacrolimus.

Actual Dose Required	N/o. of Patients (%)	Ideal Dose* (%)	Underestimation^&^	Overestimation$ (%)
Validation cohort only	126	54.8	19.8	25.4
≤2.5 mg/day	32	31.2	0	68.8
>2.5 mg/day to ≤4 mg/day	69	72.5	14.5	13.0
>4 mg/day	25	36.0	60.0	4.0
Derivation-plus-validation cohort	693	57.3	19.2	23.4
≤2.5 mg/day	169	38.5	0	61.5
>2.5 mg/day to ≤4 mg/day	380	70.5	14.4	15.0
>4 mg/day	144	44.4	54.9	0.7

^*^The ideal dose was defined as a predicted dose that was within 20% of the actual stable therapeutic dose of tacrolimus. ^&^We defined underestimation as a predicted dose that was at least 20% lower than the actual stable dose. ^$^We defined overestimation as a predicted dose that was at least 20% higher than the actual stable dose.

## References

[b1] Meier-Kriesche H. U. . Immunosuppression: evolution in practice and trends, 1994–2004. Am J Transplant 6, 1111–1131, doi: 10.1111/j.1600-6143.2006.01270.x (2006). 16613591

[b2] Wallemacq P. . Opportunities to optimize tacrolimus therapy in solid organ transplantation: report of the European consensus conference. Therapeutic drug monitoring 31, 139–152, doi: 10.1097/FTD.0b013e318198d092 (2009). 19177031

[b3] MacPhee I. A. . The influence of pharmacogenetics on the time to achieve target tacrolimus concentrations after kidney transplantation. Am J Transplant 4, 914–919, doi: 10.1111/j.1600-6143.2004.00435.x (2004). 15147425

[b4] The U.S. Multicenter FK506 Liver Study Group. A comparison of tacrolimus (FK 506) and cyclosporine for immunosuppression in liver transplantation. N Engl J Med 331, 1110–1115, doi: 10.1056/NEJM199410273311702 (1994). 7523946

[b5] Kershner R. P. & Fitzsimmons W. E. Relationship of FK506 whole blood concentrations and efficacy and toxicity after liver and kidney transplantation. Transplantation 62, 920–926 (1996). 8878385 10.1097/00007890-199610150-00009

[b6] de Jonge H., Naesens M. & Kuypers D. R. New insights into the pharmacokinetics and pharmacodynamics of the calcineurin inhibitors and mycophenolic acid: possible consequences for therapeutic drug monitoring in solid organ transplantation. Therapeutic drug monitoring 31, 416–435, doi: 10.1097/FTD.0b013e3181aa36cd (2009). 19536049

[b7] Staatz C. E., Goodman L. K. & Tett S. E. Effect of CYP3A and ABCB1 single nucleotide polymorphisms on the pharmacokinetics and pharmacodynamics of calcineurin inhibitors: Part I. Clinical pharmacokinetics 49, 141–175, doi: 10.2165/11317350-000000000-00000 (2010). 20170205

[b8] Staatz C. E., Goodman L. K. & Tett S. E. Effect of CYP3A and ABCB1 single nucleotide polymorphisms on the pharmacokinetics and pharmacodynamics of calcineurin inhibitors: Part II. Clinical pharmacokinetics 49, 207–221, doi: 10.2165/11317550-000000000-00000 (2010). 20214406

[b9] de Jonge H., de Loor H., Verbeke K., Vanrenterghem Y. & Kuypers D. R. *In vivo* CYP3A4 activity, CYP3A5 genotype, and hematocrit predict tacrolimus dose requirements and clearance in renal transplant patients. Clin Pharmacol Ther 92, 366–375, doi: 10.1038/clpt.2012.109 (2012). 22871995

[b10] Jacobson P. A. . Novel polymorphisms associated with tacrolimus trough concentrations: results from a multicenter kidney transplant consortium. Transplantation 91, 300–308, doi: 10.1097/TP.0b013e318200e991 (2011). 21206424 PMC3579501

[b11] van Gelder T. & Hesselink D. A. Dosing tacrolimus based on CYP3A5 genotype: will it improve clinical outcome? Clin Pharmacol Ther 87, 640–641, doi: 10.1038/clpt.2010.42 (2010). 20485320

[b12] Thervet E. . Optimization of initial tacrolimus dose using pharmacogenetic testing. Clin Pharmacol Ther 87, 721–726, doi: 10.1038/clpt.2010.17 (2010). 20393454

[b13] Li J. L. . Effects of diltiazem on pharmacokinetics of tacrolimus in relation to CYP3A5 genotype status in renal recipients: from retrospective to prospective. Pharmacogenomics J 11, 300–306, doi: 10.1038/tpj.2010.42 (2011). 20514078

[b14] Passey C. . Validation of tacrolimus equation to predict troughs using genetic and clinical factors. Pharmacogenomics 13, 1141–1147, doi: 10.2217/pgs.12.98 (2012). 22909204 PMC3579500

[b15] Passey C. . Dosing equation for tacrolimus using genetic variants and clinical factors. British journal of clinical pharmacology 72, 948–957, doi: 10.1111/j.1365-2125.2011.04039.x (2011). 21671989 PMC3244642

[b16] Provenzani A. . Influence of CYP3A5 and ABCB1 gene polymorphisms and other factors on tacrolimus dosing in Caucasian liver and kidney transplant patients. International journal of molecular medicine 28, 1093–1102, doi: 10.3892/ijmm.2011.794 (2011). 21922127

[b17] Li L. . Tacrolimus dosing in Chinese renal transplant recipients: a population-based pharmacogenetics study. Eur J Clin Pharmacol 67, 787–795, doi: 10.1007/s00228-011-1010-y (2011). 21331500

[b18] Kim I. W. . Clinical and genetic factors affecting tacrolimus trough levels and drug-related outcomes in Korean kidney transplant recipients. Eur J Clin Pharmacol 68, 657–669, doi: 10.1007/s00228-011-1182-5 (2012). 22183771

[b19] Wang P. . Using genetic and clinical factors to predict tacrolimus dose in renal transplant recipients. Pharmacogenomics 11, 1389–1402, doi: 10.2217/pgs.10.105 (2010). 21047202

[b20] Liu R., Li X., Zhang W. & Zhou H. H. Comparison of Nine Statistical Model Based Warfarin Pharmacogenetic Dosing Algorithms Using the Racially Diverse International Warfarin Pharmacogenetic Consortium Cohort Database. PloS one 10, e0135784, doi: 10.1371/journal.pone.0135784 (2015). 26305568 PMC4549222

[b21] Cosgun E., Limdi N. A. & Duarte C. W. High-dimensional pharmacogenetic prediction of a continuous trait using machine learning techniques with application to warfarin dose prediction in African Americans. Bioinformatics 27, 1384–1389, doi: 10.1093/bioinformatics/btr159 (2011). 21450715 PMC3087957

[b22] Klein T. E. . Estimation of the warfarin dose with clinical and pharmacogenetic data. N Engl J Med 360, 753–764, doi: 10.1056/NEJMoa0809329 (2009). 19228618 PMC2722908

[b23] Grossi E. . Prediction of optimal warfarin maintenance dose using advanced artificial neural networks. Pharmacogenomics 15, 29–37, doi: 10.2217/pgs.13.212 (2014). 24329188

[b24] Hesselink D. A. . Genetic polymorphisms of the CYP3A4, CYP3A5, and MDR-1 genes and pharmacokinetics of the calcineurin inhibitors cyclosporine and tacrolimus. Clin Pharmacol Ther 74, 245–254, doi: 10.1016/S0009-9236(03)00168-1 (2003). 12966368

[b25] Macphee I. A. . Tacrolimus pharmacogenetics: polymorphisms associated with expression of cytochrome p4503A5 and P-glycoprotein correlate with dose requirement. Transplantation 74, 1486–1489, doi: 10.1097/01.TP.0000045761.71385.9F (2002). 12490779

[b26] Kurzawski M. . CYP3A5 and CYP3A4, but not ABCB1 polymorphisms affect tacrolimus dose-adjusted trough concentrations in kidney transplant recipients. Pharmacogenomics 15, 179–188, doi: 10.2217/pgs.13.199 (2014). 24444408

[b27] Schalekamp T. . VKORC1 and CYP2C9 genotypes and phenprocoumon anticoagulation status: interaction between both genotypes affects dose requirement. Clin Pharmacol Ther 81, 185–193, doi: 10.1038/sj.clpt.6100036 (2007). 17192772

[b28] Hunter D. J. Gene-environment interactions in human diseases. Nature reviews. Genetics 6, 287–298, doi: 10.1038/nrg1578 (2005). 15803198

[b29] Cao R. & Cheng J. Deciphering the association between gene function and spatial gene-gene interactions in 3D human genome conformation. BMC genomics 16, 880, doi: 10.1186/s12864-015-2093-0 (2015). 26511362 PMC4625479

[b30] Shin J. & Cao D. Comparison of warfarin pharmacogenetic dosing algorithms in a racially diverse large cohort. Pharmacogenomics 12, 125–134, doi: 10.2217/pgs.10.168 (2011). 21174627

[b31] Laboratories B. *The R Project for Statistical Computing*, https://www.r-project.org/(2016).

[b32] Bergmeir C. & Benitez J. M. Neural Networks in R Using the Stuttgart Neural Network Simulator: RSNNS. Journal of Statistical Software 46, 1–26 (2012).22837731

[b33] Therneau T., Atkinson B. & Ripley B. *Rpart: Recursive Partitioning and Regression Trees*, https://CRAN.R-project.org/package=rpart (2015).

[b34] Ridgeway G. *Gbm: Generalized Boosted Regression Models*, https://CRAN.R-project.org/package=gbm (2015).

[b35] Meyer D. . *e1071: Misc Functions of the Department of Statistics, Probability Theory Group (Formerly: E1071), TU Wien*, https://cran.r-project.org/web/packages/e1071/ (2015).

[b36] Breiman L., Cutler A., Liaw A. & Wiener M. *Randomforest: Breiman and Cutler’s Random Forests for Classification and Regression*, https://CRAN.R-project.org/package=randomForest (2015).

[b37] Milborrow S., Hastie T., Tibshirani R., Miller A. & Lumley T. *Earth: Multivariate Adaptive Regression Splines*, https://CRAN.R-project.org/package=earth (2015).

[b38] Friedman J., Hastie T. & Tibshirani R. Regularization Paths for Generalized Linear Models via Coordinate Descent. Journal of Statistical Software 33, 1–22 (2010). 20808728 PMC2929880

[b39] Kapelner A. & Bleich J. bartMachine: Machine Learning with Bayesian Additive Regression Trees. Journal of Statistical Software 70, 1–40, doi: 10.18637/jss.v070.i04 (2016).

