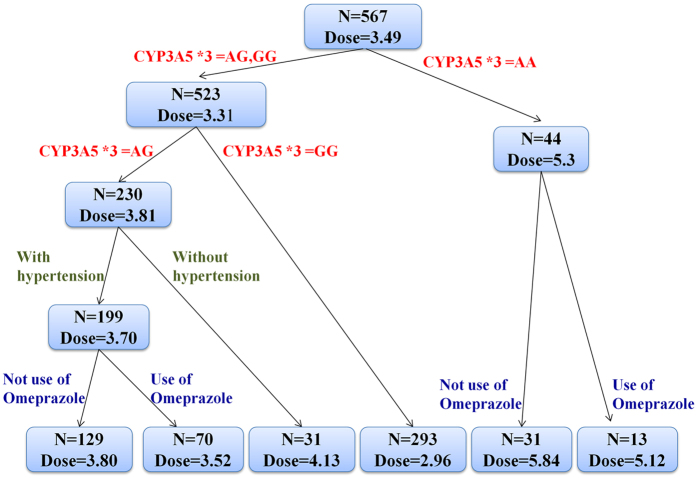# Corrigendum: Application of Machine-Learning Models to Predict Tacrolimus Stable Dose in Renal Transplant Recipients

**DOI:** 10.1038/srep46936

**Published:** 2018-01-29

**Authors:** Jie Tang, Rong Liu, Yue-Li Zhang, Mou-Ze Liu, Yong-Fang Hu, Ming-Jie Shao, Li-Jun Zhu, Hua-Wen Xin, Gui-Wen Feng, Wen-Jun Shang, Xiang-Guang Meng, Li-Rong Zhang, Ying-Zi Ming, Wei Zhang

Scientific Reports 7: Article number: 42192; 10.1038/srep42192 published online: 02 08 2017; updated: 01 29 2018.

This Article contains an error in Figure 2. For CYP3A5 *3 = AG without hypertension,

“N = 31 Dose = 3.73”

should read:

“N = 31 Dose = 4.13”

The correct Figure 2 appears below as Figure 1.

## Figures and Tables

**Figure 1:**