# Supplementary material for: Application of Machine-Learning Models to Predict Tacrolimus Stable Dose in Renal Transplant Recipients
Source: Sci Rep. 2017 Feb 8;7:42192. doi: 10.1038/srep42192 (PMC5296901; doi:10.1038/srep42192)
Supplement: Supplementary Table S1–S4 [file srep42192-s1.doc]

**Supplementary Information**

Application of Machine-Learning Models to Predict Tacrolimus Stable Dose in Renal Transplant Recipients

Jie Tang^1,2 a^, Rong Liu^1,2 a^, Yue-Li Zhang^1,2^, Mou-Ze Liu^1,2^, Yong-Fang Hu^3^, Ming-Jie Shao^4^, Li-Jun Zhu^4^, Hua-Wen Xin^5^, Gui-Wen Feng^6^, Wen-Jun Shang^6^, Xiang-Guang Meng^7^, Li-Rong Zhang^7^, Ying-Zi Ming^4^ * and Wei Zhang^1,2 *^

Table S1. Genotype frequency distribution and Hardy-Weinberg equilibrium test.

Table S2. Univariate linear regression model for tacrolimus dose.

Table S3. Multivariate linear regression model for tacrolimus dose

Table S4. Default parameters for nine algorithms

Table S5. Training and testing dataset

**Supplementary Table S1.**

| **Variable** | **Frequency (%)** | **P value of Hardy-Weinberg equilibrium test** |
| --- | --- | --- |
| CYP3A56986AG |  | 0.26 |
| A/A | 92 (9) |  |
| A/G | 408 (40) |  |
| G/G | 523 (51) |  |
| ABCB13435CT |  | 0.65 |
| C/C | 386 (38) |  |
| C/T | 489 (48) |  |
| T/T | 146 (14) |  |
| ABCB1129TC |  | 1.0×10^-5^ |
| C/C | 114 (11) |  |
| T/T | 907 (89) |  |
| ABCB11236CT |  | 0.41 |
| C/C | 122 (12) |  |
| C/T | 477 (47) |  |
| T/T | 416 (41) |  |
| ABCB12677GT |  | 1.0×10^-5^ |
| G/G | 349 (35) |  |
| G/T | 388 (39) |  |
| T/T | 264 (26) |  |

**Supplementary Table S2.**

| **Characteristic** | **Uumber of samples used** | **P value of F-test** |
| --- | --- | --- |
| ABCB13435CT | 821 | 7.31×10^-1^ |
| ABCB11236CT | 817 | 1.92×10^-1^ |
| **CYP3A56986AG** | **823** | **< 2.2×10^-16^** |
| Gender | 828 | 2.45×10^-1^ |
| Age | 813 | 6.97×10^-1^ |
| Living donor | 834 | 5.54×10^-2^ |
| Height | 587 | 3.53×10^-1^ |
| Weight | 617 | 3.86×10^-1^ |
| Hemoglobin | 612 | 1.99×10^-1^ |
| Leukocyte | 609 | 3.74×10^-1^ |
| Serum creatinine | 620 | 8.66×10^-1^ |
| Total bilirubin | 598 | 8.41×10^-1^ |
| Albumin | 490 | 3.55×10^-1^ |
| **Hypertension** | **738** | **1.73×10^-2^** |
| **Diabetes** | **690** | **4.28×10^-2^** |
| Anemia | 629 | 2.17×10^-1^ |
| Cardiac insufficiency | 523 | 8.58×10^-1^ |
| Use of Calcium channel blocker | 839 | 4.89×10^-1^ |
| Use of Metoprolol | 839 | 9.35×10^-1^ |
| **Use of Omeprazole** | **671** | **2.46×10^-2^** |
| Use of Furosemide | 839 | 7.17×10^-1^ |
| ACEI /ARA* | 839 | 2.41×10^-1^ |
| Cephalosporin | 671 | 1.99×10^-1^ |
| Infected | 671 | 8.96×10^-1^ |

**Supplementary Table S3.**

| **Vaiable** | **Beta** | **P value of F-test** |
| --- | --- | --- |
| Intercept | 5.947 | <2e-16 |
| CYP3A5*3 AG | -1.882 | <2e-16 |
| CYP3A5*3 GG | -2.673 | <2e-16 |
| Hypertension | -0.281 | 2.47×10^-2^ |
| Omeprazole | -0.183 | 2.46×10^-2^ |

**Supplementary Table S4.**

| **Algorithm** | **Parameters** |
| --- | --- |
| support vector regression | Scale=1: Per default, data are scaled internally (both x and y variables) to zero mean and unit variance  Type=eps-regression  Kernel=u'*v  Cost=1  class.weights=1  cachesize=40  tolerance=0.001  epsilon=0.1  shrinking=TRUE  cross=0  fitted=TRUE  na.action=na.omit |
| artificial neural network | Size=5  learnFuncParams=c(0.1)  maxit=50  linOut=T |
| regression tree | cp = 0.001  xval = 100  na.action: deletes all observations for which y is missing, but keeps those in which one or more predictors are missing  method="anova" |
| mulivairate linear regression | na.action=[na.omit](http://127.0.0.1:28298/library/stats/help/na.omit)  method = "qr",  singular.ok = TRUE  contrasts = NULL |
| random forest regression | mtry=3,  importance=TRUE,  na.action=na.omit |
| boosted regression tree | var.monotone=c(0,0,0),  distribution="gaussian",  n.trees=1000,  shrinkage=0.05,  interaction.depth=3,  bag.fraction=0.5,  train.fraction=0.5,  n.minobsinnode=10,  keep.data=TRUE,  verbose=FALSE,  cv.folds=3,  n.cores=1 |
| multivariate adaptive regression splines | Subset=NULL,  Weights=NULL,  wp=NULL,  na.action=na.fail,  trace=0,  glm=NULL,  degree=1,  nk: Semi-automatically calculated from the number of predictors,  thresh=0.001,  fast.k=2.0,  fast.beta=1,  linpreds=FALSE,  allowed=NULL,  pmethod="backward",  nprune=NULL,  ncross=1,  nfold=0,  stratify=TRUE,  varmod.method=0.03,  varmod.exponent=1,  varmod.conv=1,  varmod.clamp=0.1 |
| lasoo regression | Weights=1,  Offset=NULL,  Nlambda=100,  Intercept=TRUE,  Thresh=1E-7,  Exclude=none,  penalty.factor=1,  lower.limits=-Inf,  upper.limits=Inf,  maxit=10^5,  type.logistic="Newton",  type.multinomial="ungrouped" |
| Bayesian additive regression trees | Serialize=FALSE,  Seed=NULL |
